# Risk Factors of Pulmonary Hypertension in Brazilian Patients with Sickle Cell Anemia

**DOI:** 10.1371/journal.pone.0137539

**Published:** 2015-09-03

**Authors:** Clarisse Lopes de Castro Lobo, Emilia Matos do Nascimento, Renato Abelha, Ana Maria Mach Queiroz, Philippe Connes, Gilberto Perez Cardoso, Samir K. Ballas

**Affiliations:** 1 Clinical Hematology Division, Instituto de Hematologia Arthur de Siqueira Cavalcanti—HEMORIO, Rio de Janeiro, RJ, Brazil; 2 UEZO—Centro Universitário Estadual da Zona Oeste, Rio de Janeiro, RJ, Brazil; 3 Laboratory CRIS-EA 647—Section “Vascular Biology and Red Blood Cell,” University Claude Bernard Lyon 1, Villeurbanne, France; 4 Universidade Federal Fluminense (UFF), Niteroi, RJ, Brazil; 5 Cardeza Foundation, Department of Medicine, Jefferson Medical College, Thomas Jefferson University, Philadelphia, PA, United States of America; Indiana University, UNITED STATES

## Abstract

This study was a prospective cross-sectional cohort study of 125 patients with sickle cell anemia (SS) between the ages of 16 to 60 years. Enrolled patients were followed-up prospectively for 15 months. Demographic, clinical, hematological and routine biochemical data were obtained on all patients. Six-minute walk test and Doppler Echocardiography were performed on all patients. A tricuspid regurgitant jet velocity (TRJV) < 2.5 m/sec was considered normal, 2.5 ≤ TRJV ≤ 3.0 was considered mild-moderate and > 3.0 m/sec, severe. Patients with abnormal TRJV were significantly older and more anemic, had significantly higher lactate dehydrogenase (LDH) levels, reticulocyte count and incidence of death. The logistic multimodal model implemented for the 125 patients indicated that age was the covariate that influenced the outcome of normal or abnormal TRJV with a cutoff age of thirty-two years. The survival rate for the group of patients with creatinine (Cr) > 1.0 mg/dL was lower than the group with Cr ≤ 1 and normal TRJV. A coefficient matrix showed that the LDH values were weakly correlated with the reticulocyte count but strongly correlated with hemoglobin suggesting that the TRJV values were not correlated with the hemolytic rate but with anemia. Ten patients died during the follow-up of whom 7 had TRJV > 2.5 m/sec. Acute chest syndrome was the most common cause of death followed by sepsis. In conclusion, this study shows that patients with SS older than thirty-two years with high LDH, elevated TRJV, severe anemia and Cr > 1 have poor prognosis and may be at risk of having pulmonary hypertension and should undergo RHC.

## Introduction

One of the most controversial complications of sickle cell disease (SCD) in general and sickle cell anemia (SS) in particular, at the present, is pulmonary hypertension (PH). The controversy centers on determining accurate screening methods and/or identifying predicting factors, if any, of its diagnosis. Moreover, if it is properly diagnosed the controversy extends to determine the best therapeutic interventions. The words “pulmonary hypertension” like “anemia” do not indicate a specific diagnosis but imply a constellation of signs and symptoms that have many possible etiologies. The World Health Organization classifies PH into 5 groups which were collectively referred to as “pulmonary hypertension” [1, 2] with number one being pulmonary arterial hypertension (PAH). Pulmonary hypertension is defined as resting mean pulmonary artery pressure (MPAP) ≥ 25 mmHg determined by right heart catheterization (RHC). Moreover, the hallmark of PAH, besides the elevated MPAP, is a co-existent pulmonary-capillary wedge pressure ≤ 15 mmHg [3]. About 3% of patients with SS develop PAH and the overall prevalence of all types of PH in SS is approximately 6%. Although all types of PH listed in Categories 2–5 may complicate SS, the second most common type of PH in SS is category 2 which is best described as pulmonary venous hypertension (PVH) characterized by elevated level of MPAP to ≥ 25 mmHg and elevated pulmonary capillary wedge pressure to ≥15 mmHg [3–6] most commonly due to left heart disease.

Unfortunately in Brazil, we do not have this kind of statistics at the present despite the fact that there are thousands of patients with SCD in Brazil. Moreover, at HEMORIO, we follow about 3,500 active patients annually. In addition, referring selected patients to cardiology to do RHC, the gold-standard to diagnose PAH, is often rejected due to long-waiting lists for other patients with established indications for RHC. Accordingly, the aim of this study has been to establish criteria that identify patients at high risk to have PAH so that the performance of RHC can be justified. Moreover, this study prospectively focused on the clinical features of patients with abnormal TRJV.

## Materials and Methods

Adult patients provided written consent for participation in the study. Parents or legal guardians of children < 18 years old provided written consent and the children > 18 years old provided verbal assent. The study and the consent procedure were approved by the Institutional Review Board (IRB) of HEMORIO and were carried out in accordance with the Helsinki Declaration of 1975 as revised in 2008 [7]. Details of the study and its objectives were explained to all participants. Moreover, all participants were informed that they may withdraw from the study at any time without further obligation.

### Patients

The study was a prospective cross-sectional cohort study of patients with SS between the ages of 16 to 60 years. Only patients with SS or S-β^0^-thalassemia were enrolled. Exclusion criteria included history of hypertension, asthma, diabetes, cardiac disease, smoking, therapy with anti-hypertension agents, painful crises during the previous month, acute chest syndrome during the previous 3 months and blood transfusion during the previous 4 months. Announcement of the study was conducted via the distribution of brochures in the Emergency Department and the hematology clinic and by periodic announcements on the hospital paging system at HEMORIO. Patients who were interested in participating were advised to contact the investigators of the study for possible enrollment. The number of patients screened was 187. However, only 138 patients met the inclusion criteria of the study. The diagnosis of SS or S-β^0^-Thalassemia was confirmed in 125 patients (123 with SS and 2 with S-β^0^-Thalassemia). There were seventy-eight women and forty-seven men of the enrolled patients. Of the 125 patients, thirty-seven (27%) were taking hydroxyurea (HU). Eight patients with hemoglobin (Hb) SC, 2 patients with S-β^+^-Thalassemia, 2 patients with Hb SD, and one patient with Hb CC were excluded. Enrolled patients were followed prospectively over 15 months from June 1, 2009 through August 31 2010.

### Clinical and Laboratory Data

Age, sex, weight and height were obtained from all patients (Table 1). Sickle cell genotypes, Hb F and Hb A_2_ were determined by high performance liquid chromatography (HPLC). Blood counts, reticulocyte count, biochemical profile including total, direct and indirect bilirubin (TBili, DBili and IBili respectively), alkaline phosphatase (ALK), LDH, aspartate aminotransferase (AST) and serum creatinine (Cr) were determined on fasting venous blood samples by routine methods.

**Table 1:** Clinical, laboratory and echocardiographic data in patients with normal and abnormal TRJV

		TRJV < 2.5 m/sec (n = 82)	TRJV ≥ 2.5 m/sec (n = 43)	P value
**Clinical Data**				
	Patients, n	82	43	0.0007
	Age, years	27.6 ± 10.6	34.7 ± 12.9	0.0014
	Sex M/F	30/52	19/24	0.5261
	BMI kg/m^2^	20.4 ± 3.8	20.0 ± 3.1	0.5545
	6MWT, m	566.0 ± 81.4 (n = 79)	554.2 ± 109.1 (n = 39)	0.5504
	6MWT, % predicted	78.4 ± 12.1 (n = 79)	78.1 ± 18.5 (n = 39)	0.9124
	Hydroxyurea, n	33 (40.2%)	9 (20.9%)	0.0486
	Leg ulcers, n	Unknown	0	
	Priapism, n	Unknown	0	
	Deceased, n	2 (2.4%)	7 (16.3%)	0.0082
**Laboratory Data**				
	Hb, g/dL	9.0 ± 2.2	7.8 ± 1.4	0.0003
	Hct, %	25.8 ± 5.3	22.6 ± 3.8	0.0002
	WBC, 10^3^/uL	10.1 ± 3.4	10.7 ± 3.8	0.4193
	Platelets, 10^3^/uL	410.1 ± 148.4	432.0 ± 150.0	0.4374
	Hb F, %	8.7 ± 5.9	6.6 ± 5.4	0.0557
	Reticulocytes, %	10.4 ± 3.7	12.1 ± 4.5	0.0235
	BiliT, mg/dL	3.6 ± 1.8	4.5 ± 2.9	0.0752
	BiliD, mg/dL	0.6 ± 0.3	0.8 ± 0.5	0.0354
	BiliI, mg/dL	3.0 ± 1.8	3.7 ± 2.6	0.1225
	LDH, IU/L	837.0 ± 343.4	1064.8 ± 463.1	0.0059
	ALK, IU/L	126.9 ± 118.6	114.0 ± 58.1	0.4189
	AST, IU/L	44.6 ± 21.6	53.7 ± 25.3	0.0376
	Cr, mg/dL	0.8 ± 0.2	0.8 ± 0.5	0.3703
**Echocardiographic & CT Data**				
	Max PASP, mmHg	32.9 ± 3.9	40.0 ± 6.5	<0.0001
	Min PASP, mmHg	28.0 ± 3.8	35.0 ± 6.4	<0.0001
	PAMP, mmHg	14.4 ± 3.7 (n = 39)	19.4 ± 4.2 (n = 20)	<0.0001
	PA Diameter, mm	-	27.8 ± 3.4 (n = 39)	-

The numbers shown are mean ± SD for continuous variables.

Abbreviations are: 6WMT = 6 minute walk test; ALK = Alkaline Phosphatase; AST = Aspartate transaminase; TBili = Total bilirubin; DBili = Direct bilirubin; IBili = Indirect bilirubin BMI = Body mass index; Cr = Creatinine; CT = Computerized tomography scan; Hb = hemoglobin; Hb F = Fetal hemoglobin; Hct = hematocrit; LDH = **Lactate dehydrogenase**; m = meter; Max = Maximum; Min = Minimum; PA = Pulmonary artery; PAMP = Pulmonary artery mean pressure; PASP = Pulmonary artery systolic pressure; SD = Standard Deviation; sec = second; TRJV = Tricuspid regurgitant jet velocity; WBC = White blood cell count.

### Echocardiography

Examinations were performed to evaluate cardiac function using pulsed two-dimensional M mode and color flow Doppler echocardiography (HDI 5000-Vivid- E Cardiovascular Ultrasound System, GE Healthcare, Milwaukee, WI, USA). Doppler images were interpreted by a pulmonologist. Cardiac dimensions and pulmonary parameters were measured according to the criteria of the American Society of Echocardiography (Table 1) [8–10]. A tricuspid regurgitant jet velocity (TRJV) ≥ 2.5 m/sec was considered abnormal [8–10].

### Computed Tomography

Chest computed tomography (CT) was performed with high-resolution protocol in patients with TRJV was ≥ 2.5 m/sec to determine the diameter of the pulmonary artery (Table 1) after intravenous administration of non-ionic iodinated contrast medium. (Iopamidol 612mg/ml, Shering). Normal value of the pulmonary artery diameter is < 29 mm [11].

### Six-Minute-Walk Test

The Six-Minute-Walk (6MW) test has been extensively used in pulmonary and cardiovascular disorders [12, 13] as well as in SCD [14] where it reflects exercise capacity of patients and correlates with clinical severity [15]. This test was performed along a straight flat corridor with a hard surface with a walking distance of 30 meters (Table 1).

### Statistical Analysis

Several statistical analyses were performed by 2 expert statisticians using 2 different approaches. One approach used unpaired student test to compare anthropometric, biological and clinical parameters between patients with normal and abnormal TRJV. A second analysis was performed taking into account the severity of TRJV. A one-way analysis of variance (ANOVA) was used to compare patients with normal TRJV, patients with mildly-moderately elevated TRJV (TRJV ≥ 2.5 m/sec but < 3.0 m/sec) and patients with high TRJV (TRJV ≥ 3.0 m/sec). A chi-square test was also used to test the associations between TRJV and categorical covariates. To identify the independent risk factors of having elevated TRJV, a binary (normal or abnormal TRJV) or ordinal (normal, mildly-moderately or highly elevated TRJV) multivariate logistic model was used. All variables at p < 0.10 by univariate analysis were included as covariates in the multivariate regression models. Significance level was defined as p *<* 0.05. Analyses were conducted using SPSS (v. 20, IBM SPSS Statistics, Chicago, IL). Values were reported as mean ± SD as shown in Table 1 [16–19].

Another statistical approach used the Elastic Net [20] to select the variable method that identifies the highly correlated predictors. This kind of analysis is ideal when the number of predictors (P) is far greater than the number of observations (n). The appropriate model was chosen according to the outcome. After each model, a new model was implemented with the relevant variables of the previous model and so on until the final model was obtained (a model where all variables presented statistically significant association with the outcome). In addition, the nonparametric decision tree method was implemented with the same outcome and covariates regression models. Nodes in the decision trees represent random variables and branches define directed dependencies quantified by probability distributions. The nonparametric tree method is based on a decision rule approach, implemented with a theory of conditional inference procedures and selection of variables. The tree is aimed at reducing the impurity degree by finding the point that provides greater homogeneity (higher probability of purity) inside a node and greater heterogeneity between nodes. The 5% significance level was adopted for the entire study. For the implementation of decision trees the party package [18] of the R software [17] was used.

The correlation coefficient matrix was performed to measure the association between several parameters: age; Hb; reticulocytes; LDH; and IBili. The R software was used for this statistical approach.

## Results

**Fig 1:**
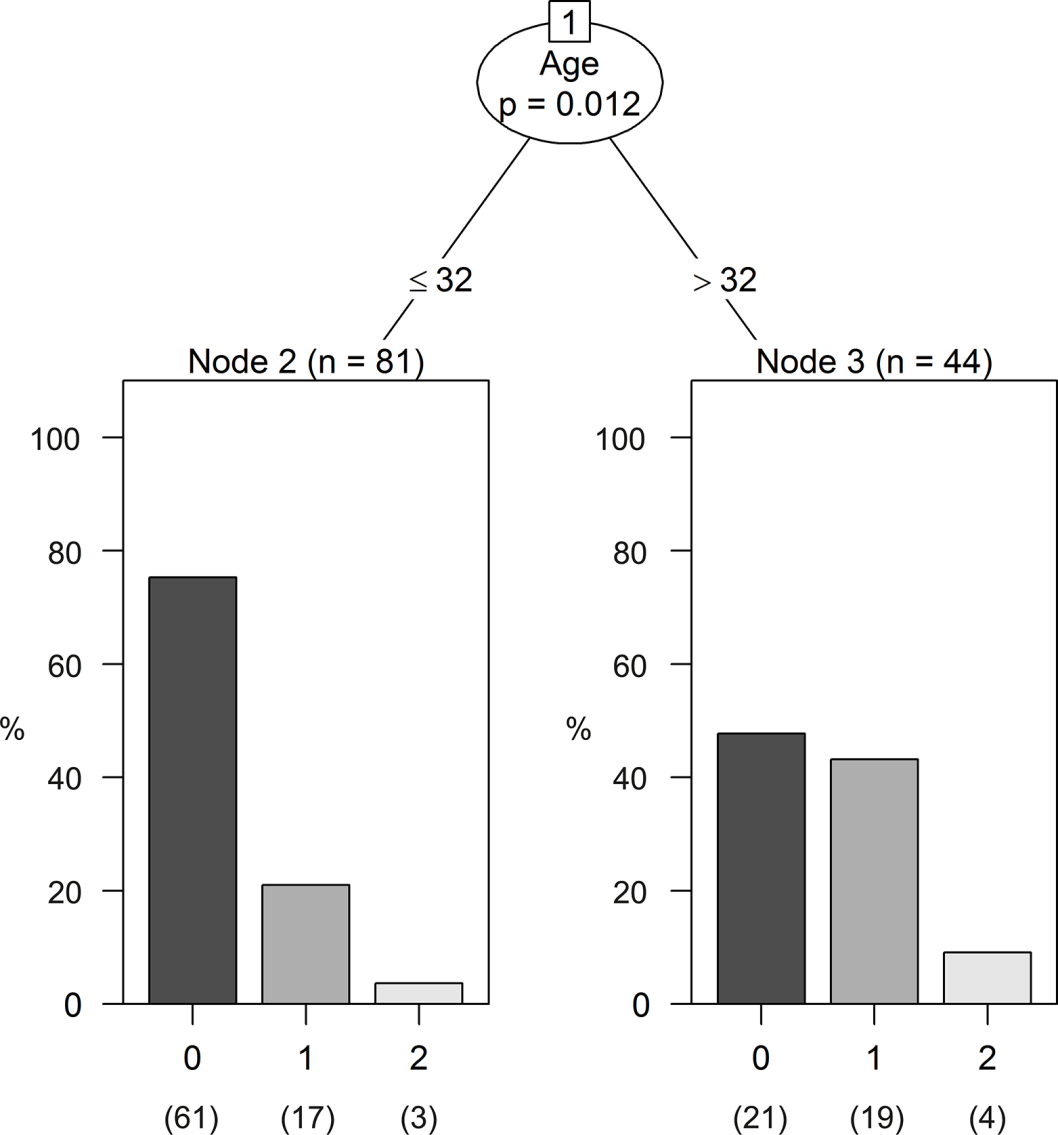
Classification tree showing bar plots in each terminal node. Each node represents a group of individuals with similar characteristics. Node 2 shows that 61 patients (75%) below the age of 32 have normal TRJV, 17 (21%) have mild-moderately severe TRJV and 3 (4%) have severely abnormal TRJV. Node 3 shows that among patients who are older than 32 years, 21(48%) have normal TRJV, 19 (43%) have mild-moderately severe TRJV and the remaining 4 patients (9%) have severely abnormal TRJV.

Of the 125 patients enrolled in the study forty-seven (38%) were men and seventy-eight (62%) were women. Tricuspid regurgitant jet velocity measurements were done on all patients: forty-three (34%) patients had abnormal TRJV while eighty-two (66%) patients exhibited normal TRJV. Table 1 lists the clinical, laboratory and echocardiographic data determined in both groups. Patients with abnormal TRJV were significantly older (p = 0.0014), significantly more anemic (p = 0.0003), had significantly higher LDH levels (p = 0.00 59), higher reticulocyte count (p = 0.0235) and higher incidence of death (p < 0.0082). The unconjugated bilirubin was not statistically different between the 2 groups (p > 0.05) suggesting the absence of hyperhemolysis. Moreover, the reticulocyte count of the patients with TRJV ≥ 2.5 m/sec was not high enough to indicate the presence of hyperhemolysis given that thirty-three patients with normal TRJV were taking HU, which inhibits erythropoiesis and decreases the reticulocyte count thus maximizing the difference between the 2 groups of patients. Fetal Hb tended to be (0.05 > p < 0.10) higher in the group with normal TRJV. All other parameters shown in Table 1 were not significantly different between the 2 groups (p > 0.05). The logistic multimodal model implemented for the 125 patients indicated that age (p = 0.002) was the covariate that influenced the outcome of normal or abnormal TRJV. The classification tree shown in Fig 1 reinforces this result with a cutoff age of thirty-two years.

**Fig 2:**
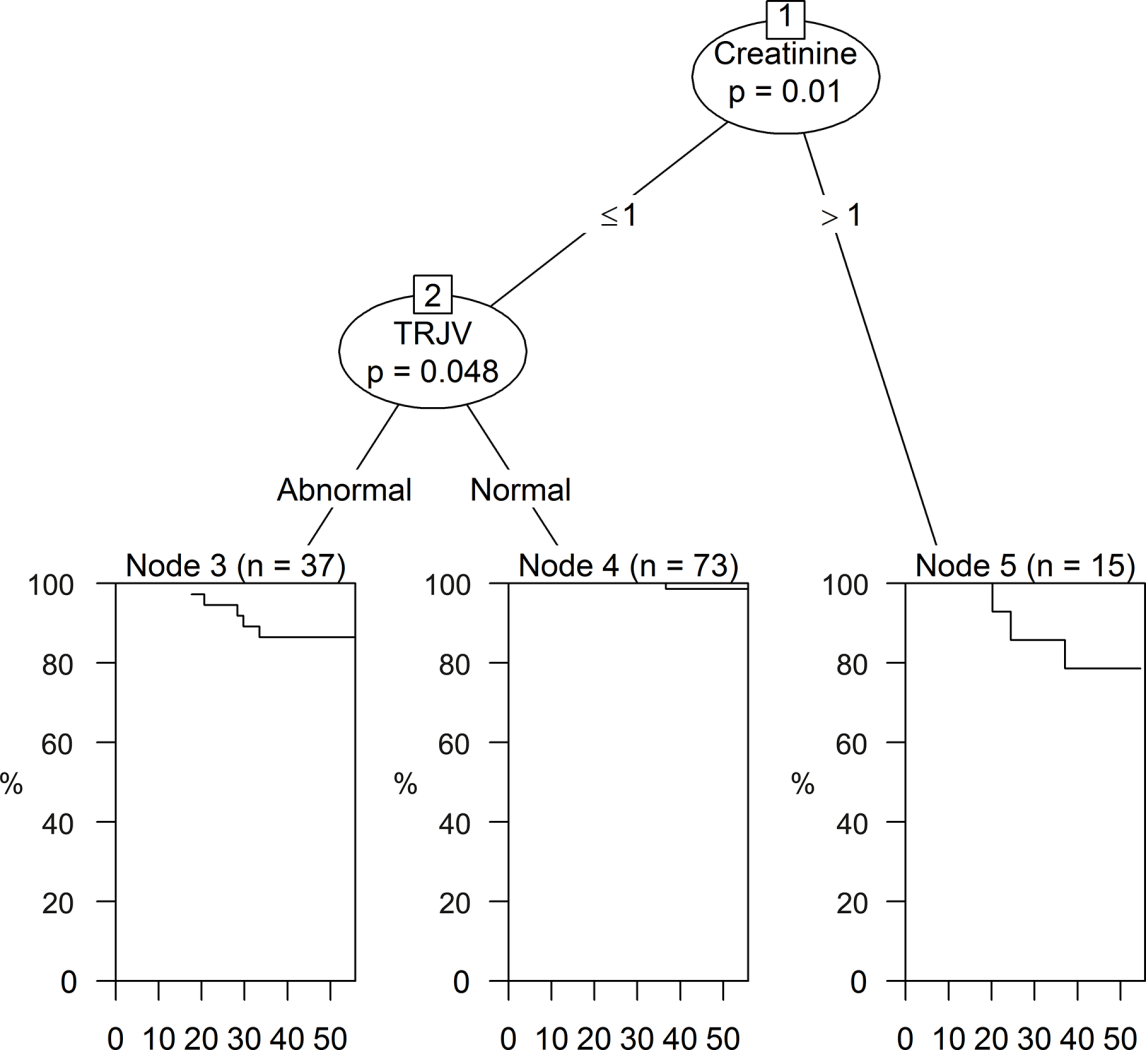
Survival tree showing a Kaplan-Meier curve in each terminal node. Patients with creatinine ≤ 1 are divided into two groups. The first group (node 3) includes 37 patients with abnormal TRJV and the second group (node 4) includes 73 patients with normal TRJV. Node 5 represents a group of 15 individuals with creatinine > 1. The X axis of all nodes shows the follow up time in months and the Y axis represents the survival probability. Thus, Node 3 shows that the probability of survival for 50 months of follow up is about 85%, in Node 4 it is 100% and in Node 5 it is about 80%. The 73 individuals in node 4 with normal TRJV have better survival probability than patients in nodes 3 and 5.

The Cox model [16] identified no variable with statistical significance (data not shown). However, the survival tree (Fig 2) identified relevant variable for Cr (p = 0.01) with a cutoff value of 1and an interaction with TRJV. The survival rate for the group of patients with creatinine greater than 1 was lower than the group with Cr ≤ 1 and normal TRJV values.

**Fig 3:**
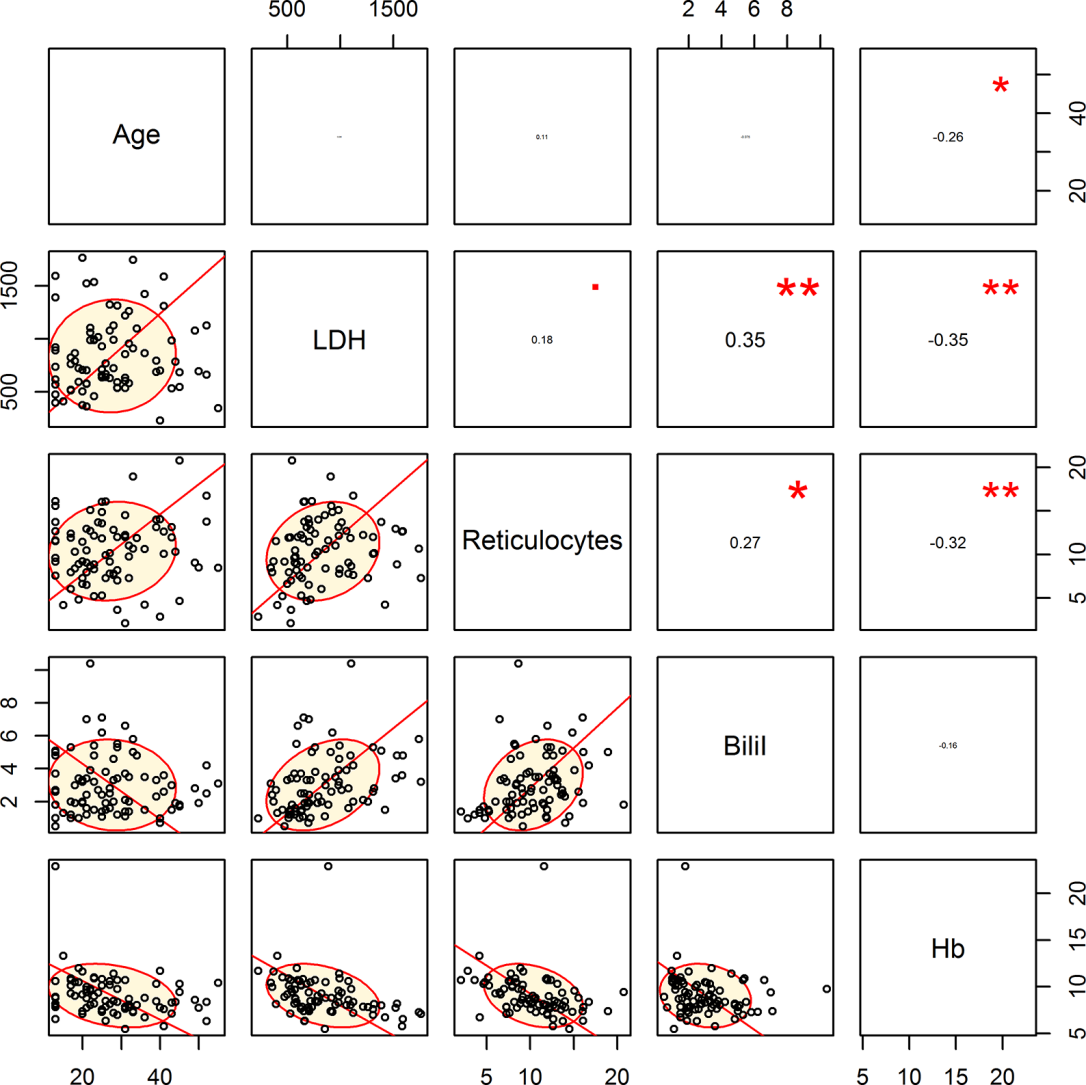
Correlation plot showing low correlation between each pair of variables for the eighty-two patients with normal TRJV (TRJV<2.5). The correlation between LDH and indirect bilirubin (r = 0.35) is slightly more than indirect bilirubin and reticulocytes (r = 0.27). However it is stronger than the correlation between LDH and reticulocytes (r = 0.18).

**Fig 4:**
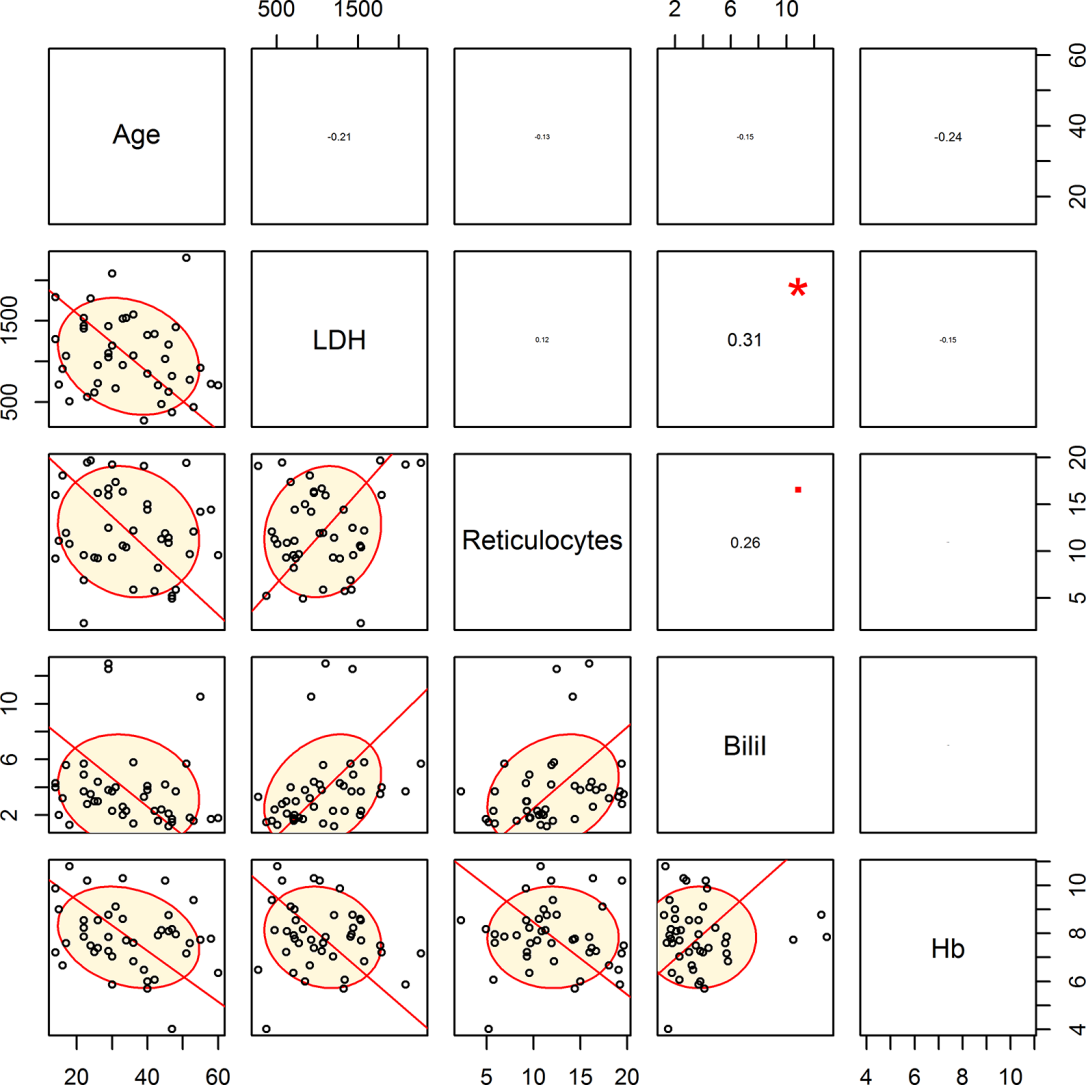
Correlation plot shows low correlation between each pair of variables in the forty-three patients with abnormal TRJV (TRJV ≥2.5). The correlation between LDH and indirect bilirubin (r = 0.31) is slightly more than indirect bilirubin and reticulocytes (r = 0.26). However it is stronger than the correlation between LDH and reticulocytes (r = 0.12).

The data was analyzed further by designing a coefficient matrix (Figs 3 and 4 and Table 2) showing that the variables were weakly correlated with each other both in patients with normal or abnormal TRJV. Fig 3 shows the coefficient matrix of the eighty-two patients with normal TRJV. The correlation between LDH and IBili (r = 0.35) is slightly higher than IBili and reticulocytes (r = 0.27) but is stronger than the correlation between LDH and reticulocytes (r = 0.18). The LDH values were weakly correlated with the reticulocyte count but strongly correlated with Hb, suggesting that the TRJV values are not correlated with the hemolytic rate but with the anemia per se whether hemolytic or not. Similarly, Fig 4 shows the coefficient matrix of the forty-three patients with abnormal TRJV. Again, the correlation plot shows poor correlation between each pair of variables. The correlation between LDH and IBili (r = 0.31) is slightly higher than IBili and reticulocytes (r = 0.26) but is stronger than the correlation between LDH and reticulocytes (r = 0.12). Moreover, comparing the data of the normal and abnormal correlation plots showed no significant difference (P >0.05) between the 2 independent correlations (Table 2). Together the data suggests that the changes observed in patients with abnormal TRJV are due to the anemia per se and not to the hemolytic rate.

**Table 2:** Correlation Coefficients between each of two variables in patients with normal and abnormal TRJV.

Correlations			
	Normal TRJV (n = 82)	Abnormal TRJV (n = 43)	P
Age & LDH	0.04	-0.21	0.19
Age & Reticulocytes	0.11	-0.13	0.21
Age & IBili	-0.08	-0.15	0.71
Age & Hb	-0.26	-0.24	0.91
LDH & Reticulocytes	0.18	0.12	0.75
LDH & IBili	0.35	0.31	0.82
LDH & Hb	-0.35	-0.15	0.27
Reticulocytes & IBili	0.27	0.26	0.96
Reticulocytes & Hb	-0.32	0.00	0.09
IBili & Hb	-0.16	0.00	0.41

Normal: TRJV<2.5 m/sec; Abnormal: TRJV≥2.5 m/sec.

Hb = hemoglobin; IBili = indirect bilirubin; LDH = lactate dehydrogenase; TRJV = tricuspid regurgitant jet velocity.

Ten patients died during the follow-up period. One of these deaths was not related to SS but to a motor vehicle accident and, hence, was excluded from statistical analyses. Table 3 lists the characteristics of the deceased patients whose cause of death is related to SCD. Seven of the deceased patients had TRJV > 2.5 m/sec and 2 had TRJV < 2.5 ms^-1^. Acute chest syndrome was the most common cause of death followed by sepsis.

**Table 3:** Cause of death and patient characteristics.

Patient	Sex	Age yrs	TRJV m/sec	$\bar{P}\mathrm{pa}$ mmHg	ECO PASP MAX	ECO PASP MIN	Cause of death
1	M	40	2.7	N	35	35	Acute chest syndrome
2	M	34	2.6	N	36	31	Acute chest syndrome
3	M	60	2.5	20	34	29	Acute chest syndrome
4	F	36	2.4	N	33	28	Acute chest syndrome
5	M	18	3	16	46	41	Stroke
6	F	40	2.9	N	39	39	Sepsis
7	F	29	0	12	36	31	Unknown
8	F	42	2.6	N	38	33	Sepsis
9	F	50	2.4	17	34	34	Acute chest syndrome
10	M	55	2.8	N	41	36	Sepsis

F: female; M: male; TRJV: tricuspid regurgitant jet velocity; $\bar{P}\mathrm{pa}$: mean pulmonary artery pressure; ECO PSAP: Echocardiographic pulmonary artery systolic pressure; m = meter; Max: Maximum; Min: Minimum; sec = second.

## Discussion

Sickle cell disease in general and SS in particular are common in Brazil. A report in 2007 indicated that SS is the most common monogenic disease in Brazil [21]. The actual number of patients, however, is unknown but is estimated by the Brazilian ministry of health to be about 25,000–30,000 patients and approximately 3,500 new patients with SS are diagnosed annually [21]. The improvement in the clinical management in the past 2–3 decades prolonged the survival of patients at HEMORIO resulting in a significant increase in the number of patients older than forty-five years. With longer longevity it is expected that a new host of co-morbidities and new complications of SCD will become apparent.

Similar to previous studies [1, 3, 22, 23] our findings show that abnormal TRJV is associated with poor prognosis and high mortality rate among patients with SS. Nine (7.2%) of the patents in the study died and the major cause of death was ACS as it is in non-Brazilian populations with SS. Although an abnormal TRJV is known to be associated with increased mortality in patients with SS, there is no evidence of association between TRJV and specific complications of SS such as ACS, sepsis and stroke. Doppler Echocardiography, is also not accurate in predicting PAH as was shown in previous studies [3, 24]. Accordingly, it is most likely that the majority of our patients with abnormal TRJV would not have PAH. Our plan is to follow these twenty-three patients (Fig 1) with abnormal TRJV aged > thirty-two years who seem to be at high risk for having PAH.

**Fig 5:**
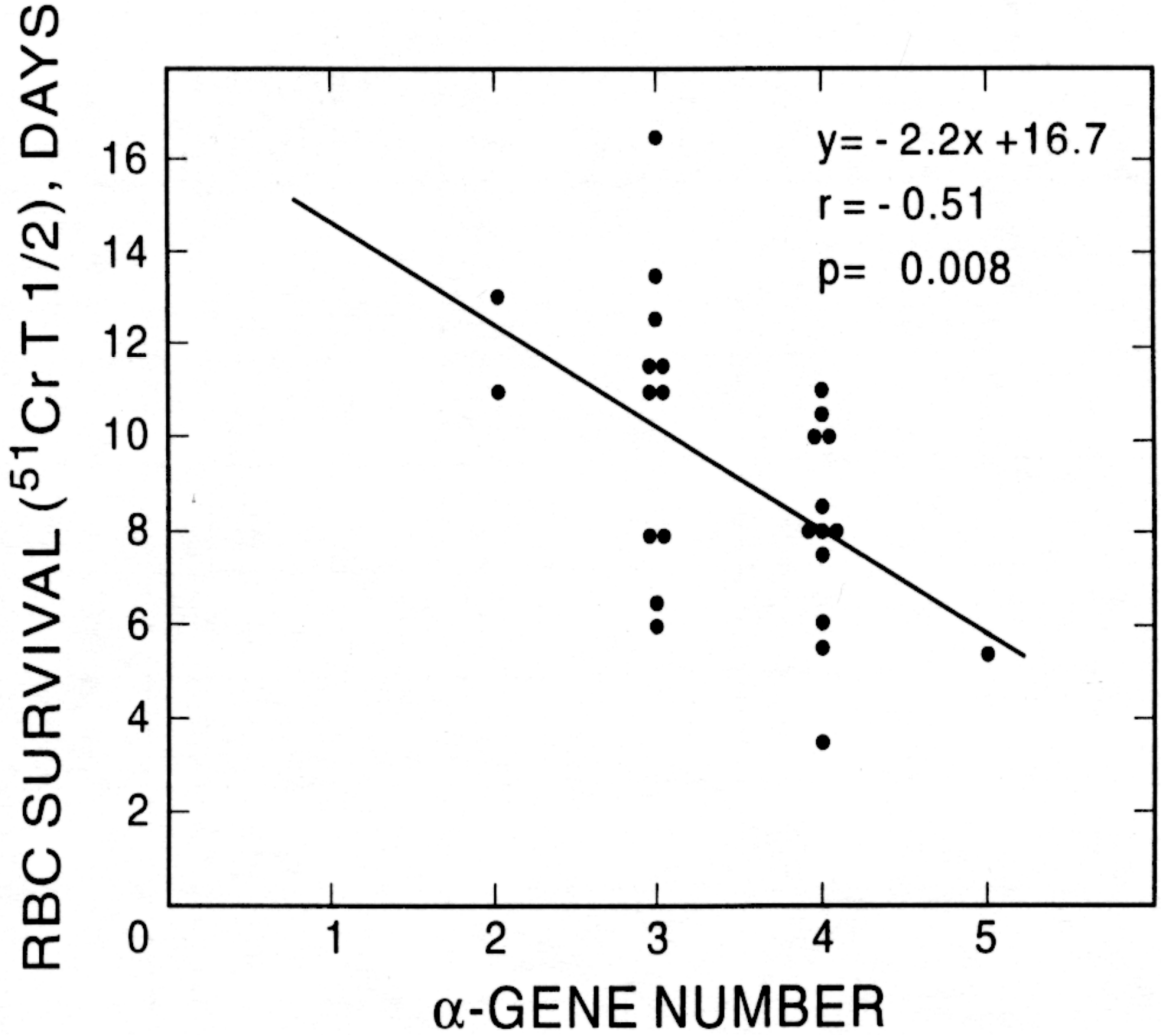
Effect of α genotypes on RBC survival in patients with sickle cell anemia in the steady state. From Ballas and Marcolina, Hemoglobin 24:277, 2000 with permission.

**Fig 6:**
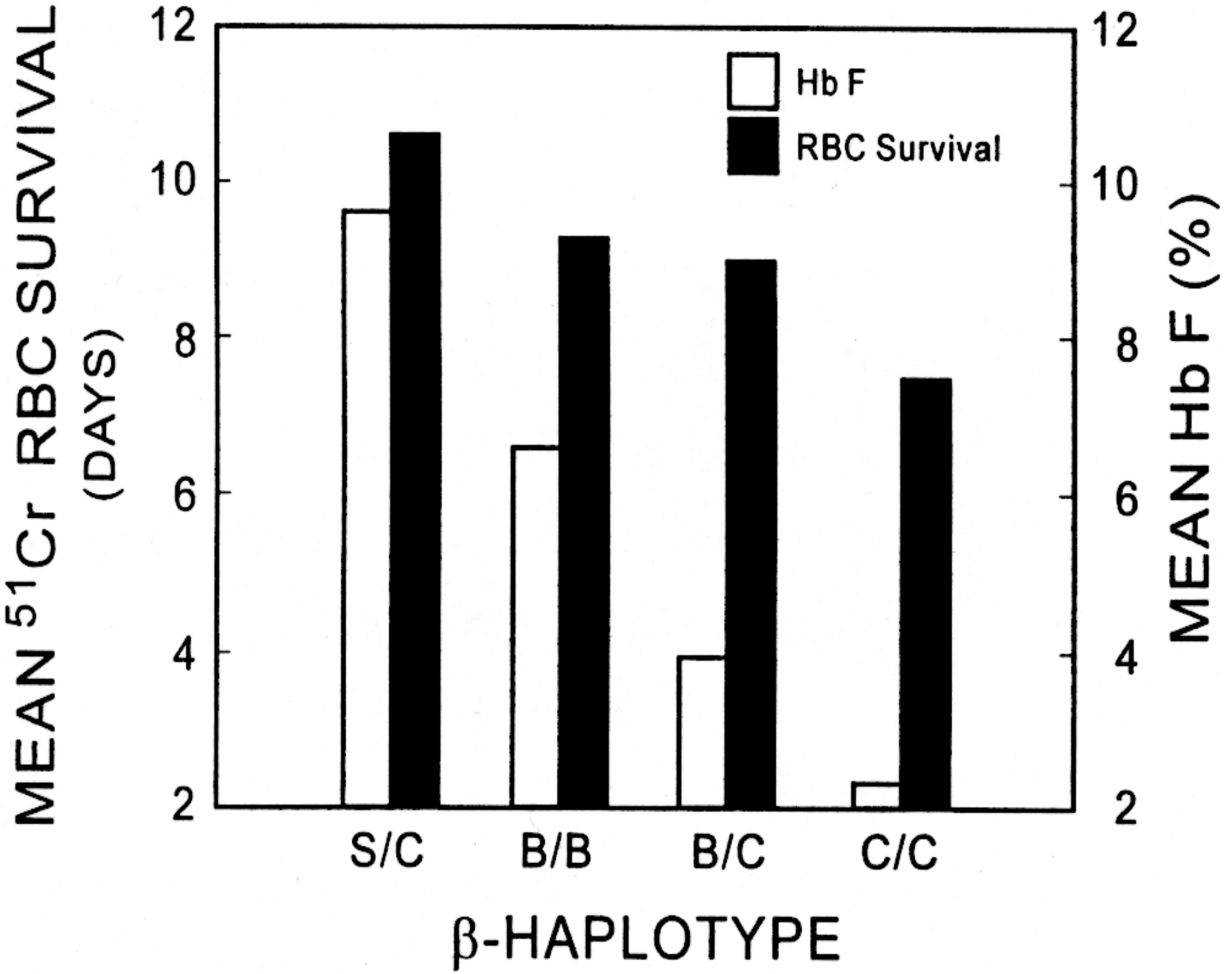
Effects of Hb F and β-Haplotypes on RBC survival in patients with sickle cell anemia in the steady state. From Ballas and Marcolina, Hemoglobin 24:277, 2000 with permission. S: Senegal β haplotype; C: Central African Republic (also known as Bantu) β haplotype and B: Benin β haplotype.

Patients with SS who are older than thirty-two years of age with abnormal TRJV, high LDH and severe anemia are at risk of having PH. It is important to note that the anemia may not exclusively be due to hyperhemolysis. The latter term is often loosely used and based on the assumption that the LDH level is a measure of red cell survival. There is no evidence for this assumption [25]. Sickle cell anemia is a hemolytic red cell disorder and all patients with SCD have variable degrees of hemolysis and it is most severe in SS. The severity of hemolysis in SS varies among patients depending on Hb F level, β^s^ haplotypes and the presence or absence of co-existent α thalassemia as shown in Figs 5 and 6 [26]. The LDH enzyme is ubiquitous and found in almost all organs in the body [25]. Tissue damage due to surgery, trauma or other acute or chronic disease is usually associated with increased levels of LDH. High levels of LDH in multiple myeloma, for example, are associated with disease severity and poor prognosis although hemolysis is not an issue in this disease [27, 28]. The parameters of hemolysis were determined on all patients enrolled in the Multicenter Study of Hydroxyurea (MSH) in SS and LDH was not one of them [29–31]. In true hyperhemolysis the Hb level decreased from an average of 8.5 ± 1.94 to an average of 5.8 ± 1.12 g/dL (p<0.001), the reticulocyte count increased from an average of 9.5 ± 5.44 to an average of 20.9 ± 8.14% (p <). 001), the RBC ^51^Cr t_1/2_ survival decreased from an average of 13.4 ± 3.78 days to 8.0 ± 3.06 days (p = 0.034), the total cell life decreased from an average of 35.4 ± 14.23 days to an average of 16.6 ± 8.77 days (p = 0.033) and the RBC Hb/reticulocyte Hb ratio decreased from an average of 17.0 ± 6.3 to an average of 7.5 ± 1.89 (p < 0.001) [32, 33]. Thus, in our study the modest absolute increase in reticulocyte count and IBili do not seem to be high enough to be due to hyperhemolysis but, rather, manifestations due to the ongoing hemolysis characteristic of SS. This overutilization of the diagnosis of hyperhemolysis becomes clear in an excellent study by Fonseca et al. of PH diagnosed by RHC in SCD [34]. The authors indicated that the patients with proven PH by RHC had hyperhemolysis due to significantly higher levels of LDH. The data listed in the tables of the study showed that the reticulocyte count and the IBili levels, two true markers of hemolysis, were not significantly different between the 2 groups of the study. The Hb level, however, was significantly lower in patients with proven PAH compared to the group with negative RHC findings. Thus it is the severity of the anemia irrespective of the rate of hemolysis that is associated with PAH.

Abnormal kidney function is an important parameter to monitor in patients at risk to develop PAH. Ataga et al. [35] found that albuminuria is associated with PH in patients with SCD. The association seems to be due to soluble fms-like tyrosine kinase-1 that may play a role in linking glomerulopathy to the endothelial dysfunction in patients with PH and SCD [35]. Similarly, our patients who had Cr level > 1.0 mg/dL had higher mortality and a small number of patients with Cr level < 1.0 mg/dL and abnormal TRJV also had higher mortality rate. The study by Fonseca et al. [34] found that patients with SCD and proven PAH by RHC had significantly higher levels of blood urea nitrogen and Cr.

The weakness of the study was that there was no matched group of normal Brazilian individuals. The absence of RHC data at the present time for high risk patients is another weakness of the study. The strengths of this study, however, include the lack of bias in selecting patients for enrollment and the fact that the study was prospective in nature. The enrolled patients volunteered to be in the study by responding to the brochures and announcements about the study. More patients responded for enrollment than expected. Moreover, there were no drop-outs from the study during the 15 months of follow-up.

In summary, our study found that the following criteria indicated a possible risk of PAH and justifies further testing with RHC in patients with SS: 1) thirty-two years of age or older; 2) who have severe anemia which is not necessarily due to hyperhemolysis; 3) abnormal TRJV; 4) elevated LDH levels due to tissue damage; and 5) Cr level > 1.0 mg/dL. Despite the limited availability in Brazil, these patients should be considered for RHC to evaluate the diagnosis of PAH and initiate appropriate therapy as needed.
